# Political and social determinants of life expectancy in less developed countries: a longitudinal study

**DOI:** 10.1186/1471-2458-12-85

**Published:** 2012-01-27

**Authors:** Ro-Ting Lin, Ya-Mei Chen, Lung-Chang Chien, Chang-Chuan Chan

**Affiliations:** 1 Institute of Occupational Medicine and Industrial Hygiene, College of Public Health, National Taiwan University, Room 722, No. 17, Xuzhou Road, Taipei City 100, Taiwan; 2 Department of Internal Medicine, Division of Health Behavior Research, School of Medicine, Washington University in St. Louis, 4444 Forest Park Avenue, Suite 6700, St. Louis 63108, USA; 3 Global Health Center, College of Public Health, National Taiwan University, No. 17, Xuzhou Road, Taipei City 100, Taiwan

**Keywords:** Life expectancy, Socioeconomic factors, Developing countries, World health, Political factors, Public health, Malnutrition, Literacy, Democracy

## Abstract

**Background:**

This study aimed to examine the longitudinal contributions of four political and socioeconomic factors to the increase in life expectancy in less developed countries (LDCs) between 1970 and 2004.

**Methods:**

We collected 35 years of annual data for 119 LDCs on life expectancy at birth and on four key socioeconomic indicators: economy, measured by log10 gross domestic product per capita at purchasing power parity; educational environment, measured by the literacy rate of the adult population aged 15 years and over; nutritional status, measured by the proportion of undernourished people in the population; and political regime, measured by the regime score from the Polity IV database. Using linear mixed models, we analyzed the longitudinal effects of these multiple factors on life expectancy at birth with a lag of 0-10 years, adjusting for both time and regional correlations.

**Results:**

The LDCs' increases in life expectancy over time were associated with all four factors. Political regime had the least influence on increased life expectancy to begin with, but became significant starting in the 3rd year and continued to increase, while the impact of the other socioeconomic factors began strong but continually decreased over time. The combined effects of these four socioeconomic and political determinants contributed 54.74% - 98.16% of the life expectancy gains throughout the lag periods of 0-10 years.

**Conclusions:**

Though the effect of democratic politics on increasing life expectancy was relatively small in the short term when compared to the effects of the other socioeconomic factors, the long-term impact of democracy should not be underestimated.

## Background

The extension of life expectancy has always been a primary interest of medical research as well as an indicator of national public health profiles [1]. Life expectancy has exhibited patterns of continuous growth over time, but it has also demonstrated persistently high variability between countries over the past half-century [2,3]. As of 2008, the gap in life expectancy between regions classified by the United Nations (UN) as more developed and less developed was as high as 11 years [4].

Changes in life expectancy can result from long-term changes in many factors, including political regime and socioeconomic status [5,6]. Political regime has been used as a distal determinant of life expectancy at the country level [7,8]. A more democratic country may more readily recognize citizens' rights to voice and act on political opinions, and therefore may produce public services that are more closely tied to social needs [9,10]. Under electoral incentives, politicians govern public policies on labor market and welfare issues to avoid famine, to increase per capita income, to increase public health and medical care expenditures, and to improve the health and quality of life of the population [8,10]. For example, labor market policies that promote higher employment rates and salary levels could contribute to better economic status and population health [8]. Furthermore, investment in welfare and health policies--such as ensuring safe childbirth for mothers and babies, securing children's right to nutrition, enhancing education of women and children, and increasing accessibility of public health and medical services--could benefit population health by redistributing resources to more people who are in need [8,11,12].

There has been growing interest in the concept of political empowerment and related health outcomes [13-15]. Powerlessness, or the lack of control over one's destiny, may be a broad-based risk factor for disease. Empowerment can be demonstrated to be an important promoter of health [16]. Some studies have shown that people who live in more democratic societies, which were assumed to empower people with more autonomy, have longer life expectancies and lower mortality rates than do people who live in more autocratic societies; other studies have shown that democracy has little or no effect on mortality rates among the poor [7,8,17]. For example, South Africa became a representative democracy in 1994, but it has shown worsening health indicators ever since [18]. Reviews of the influence of democracy on population health over time have not only been intriguing [10], but have hypothesized and proven that democracy has real and important effects on the daily lives and well-being of individuals around the globe [8,10].

However, the influence of political regime and socioeconomic factors on life expectancy has yet to be studied comprehensively, and analysis of the long-term effects of political regime is particularly lacking. By nature, a time lag exists between policy design and the full effect of the policy [17,19]. Even if a changing political regime initiates immediate changes to public services, the level of public services produced by the state will take time to change significantly. The lack of comprehensive studies has been mainly due to the limitations of short study time frames and the scarcity of comparable data [7]. These limitations may have contributed to the inconsistent research findings regarding democracy and life expectancy. Study design could be another factor contributing to the inconsistent findings. Previous studies investigating social and policy determinants' long-term effects on health outcomes on a global scale used regression analyses and data from a single time point to predict health outcomes at a single time point [10]. Such design is subject to the influence of global socioeconomic changes: the findings may vary depending on the socioeconomic changes in the world during that specific period of time [7,8,10]. Moreover, regression techniques may ignore within-country correlations when longitudinal data are modelled, and thus lead to biased estimates of regression parameters and results [20]. Other designs, such as time series analyses, may drive a better estimation of the association between time-varying determinants and the longitudinal trend of life expectancy. For this study, publicly available country-specific long-term data on life expectancy and political and socioeconomic factors enabled us to address these important issues through longitudinal data modelling.

This study aimed to investigate the longitudinal relationships between life expectancy and national developments in political regime in less developed countries (LDCs). Life expectancy at birth was the outcome variable. Life expectancy at birth reflects the overall mortality rate of a population with consideration of infant and child mortality, which are susceptible to both political and socioeconomic risk factors [7,8]. The inclusion of child health is also important because it can reflect public health policies and efforts against infectious diseases and malnutrition [8,21].

In addition to political regime, several main socioeconomic indicators found to be important determinants of life expectancy, such as economy, educational environment, and nutritional status, were also included for investigation [22-24]. Variations in life expectancy across countries have been attributed, in cross-sectional studies, to increases in national income (by 10% - 25%) and literacy (by 59% - 64%), after controlling for the state of the economy and the level of income inequality [23,24]. Poor nutritional status affects mothers and children in countries with low incomes and accounts for 11% of the global disease burden [21,25-27].

Unlike the studies which examined data from only one time point to predict health effects in the future, we examined the lagged effects of the selected factors on life expectancy at birth across a period of 35 years from 1970 to 2004. We adjusted for time and regional correlations in order to determine whether and how changes in life expectancy are the result of changes in the selected socioeconomic factors over time. To address these issues, we first present the longitudinal relationships between life expectancy in LDCs and the respective socioeconomic factors, and then illustrate the modelling results and estimations regarding the impact of each factor on life expectancy in LDCs between 1970 and 2004. Understanding from a longitudinal perspective how political regime and these multidimensional socioeconomic factors contribute to increased life expectancy could provide further evidence to support global health efforts, especially for developing countries [28,29].

## Methods

We applied two classifications in selecting countries to be included as LDCs in this study. First, we used the classification employed by the United Nations (UN). We identified as LDCs 169 countries from regions classified by the UN as less developed, including Africa (N = 57), Asia (with the exception of Japan) (N = 42), Latin America and the Caribbean (N = 48), and Oceania (with the exception of Australia and New Zealand) (N = 22). Second, we followed the criteria for the "Developed World" category defined by the United States Census Bureau (USCB). We classified as LDCs those countries not included in the USCB's "Developed World" category [30]. Therefore, 20 Eastern and Southern European countries (with the exception of Italy) and 15 newly independent countries from the former Union of Soviet Socialist Republics were also identified as LDCs for this study. Out of these 204 total LDCs, 119 countries, representing 83.28% of the world's population from 2000-2004, had at least 1 year's worth of data available for analysis. These 119 countries with available data were included for analysis and are listed in Table 1.

**Table 1:** Countries included in the analysis, by geographical region^a ^(N = 119)

Regions	N	Countries
**Africa**	**45**	
Eastern Africa	15	Burundi, Comoros, Djibouti, Eritrea, Ethiopia, Kenya, Madagascar, Malawi, Mauritius, Mozambique, Rwanda, Tanzania, Uganda, Zambia, and Zimbabwe
Middle Africa	6	Cameroon, Central African Republic, Chad, Congo, Democratic Republic of the Congo, and Gabon
Northern Africa	5	Algeria, Egypt, Morocco, Sudan, and Tunisia
Southern Africa	5	Botswana, Lesotho, Namibia, South Africa, and Swaziland
Western Africa	14	Benin, Burkina Faso, Côte d'Ivoire, Gambia, Ghana, Guinea, Guinea-Bissau, Liberia, Mali, Mauritania, Niger, Nigeria, Senegal, and Togo
**Oceania**	**1**	Fiji
**Asia**	**26**	
Eastern Asia	3	China, Mongolia, and Republic of Korea
Southern Asia	6	Bangladesh, India, Iran, Nepal, Pakistan, and Sri Lanka
South-Eastern Asia	7	Cambodia, Indonesia, Laos, Malaysia, the Philippines, Thailand, and Vietnam
Western Asia	10	Cyprus, Israel, Jordan, Kuwait, Lebanon, Saudi Arabia, Syria, Turkey, United Arab Emirates, and Yemen
**Latin America and the Caribbean**	**23**	
Caribbean	5	Cuba, Dominican Republic, Haiti, Jamaica, and Trinidad and Tobago
Central America	7	Costa Rica, El Salvador, Guatemala, Honduras, Mexico, Nicaragua, and Panama
South America	11	Argentina, Bolivia, Brazil, Chile, Colombia, Ecuador, Guyana, Paraguay, Peru, Uruguay, and Venezuela
**Southern and Eastern Europe**	**12**	
Eastern Europe	4	Bulgaria, Hungary, Poland, and Romania
Southern Europe	8	Albania, Croatia, Greece, Macedonia, Portugal, Serbia and Montenegro, Slovenia, and Spain
**Eurasia**	**12**	Armenia, Belarus, Estonia, Kazakhstan, Latvia, Lithuania, Moldova, Russia, Tajikistan, Turkmenistan, Ukraine, and Uzbekistan

N = number of countries. ^a^Based on the United Nations' geographical regions

### Data and measures

We obtained data for each of the 119 LDCs on historical life expectancy at birth and on indicators of socioeconomic and political status over a 35-year period, 1970-2004, all from publicly available databases. The year-by-year life expectancy of each nation was based on interpolated demographic indicators included in information from the UN World Population Prospects and the United States Census Bureau (USCB) [4,31].

The study investigated the following factors as determinants of life expectancy: economy, literacy, undernourishment, and political regime. Political regime was measured by a score that reflected the extent of democratic and autocratic authority patterns institutionalized in the country during its political lifespan [32]. The score used to measure the characteristics of a country's political regime was the POLITY2 variable from the Polity IV dataset. Polity IV was a project to measure longitudinal patterns of political characteristics and contestability for states in the world system [32]. In order to facilitate the use of the regime measure in time-series analyses, the Polity IV project modified a previously categorical score into continuous score--the POLITY2 score [32]. The variable was transferred as a continuous variable either by scoring anarchy as zero or by prorating the score over the transition span for countries experiencing authority change [32]. The POLITY2 variable took into account and was weighted based on dimensions of openness and competitiveness in recruitment of political actors as well as on constraints on chief political executives [32]. The regime status was then scored on a spectrum ranging from -10 (strongly autocratic) to +10 (strongly democratic) [32]. The POLITY2 score was treated as a continuous variable for analysis in this and previous studies [9,32].

The measure used to indicate the state of each country's economy was calculated from the level of per capita income based on the yearly per capita gross domestic product (GDP) at purchasing power parity, in current prices. These data were extracted from the Penn World Table [33]. Literacy, measured as the proportion of the country's population over the age of 15 years who were literate, was used as a measure for educational environment. Literacy data were retrieved primarily from UN agencies [34,35]. Undernourishment was used as a proxy for nutritional status and was measured by calculating the proportion of each country's population whose dietary condition was continuously below a minimum dietary energy requirement. These data were extracted from UN Food and Agriculture Organization statistics [36]. For the purposes of our calculations, countries in which less than 2.5% of the population was undernourished had the relevant metric set to 2.5% [36].

### Statistical methods

This study used the general linear mixed model to investigate the lagged effects of the political regime and socioeconomic factors on life expectancy while controlling both for autoregressive correlations over time and for regional correlations. We performed a PROC MIXED procedure using the SAS V9.1.3 software package (SAS Institute Inc., Cary, NC, USA) to analyze the longitudinal effects of the selected factors on life expectancy from 1970 to 2004. The life expectancy (Y) of a country was the dependent variable. Five fixed effects--time (T), the common logarithm of the economy (E), the literacy rate (L), the proportion of the population undernourished (U), and the political regime score (P)--were taken as the independent variables. We applied linear mixed models [37-39] in which regional heterogeneous covariance were considered, as follows:

Yi(k),j = b0i(k) + β0 + β1 × Ti(k),j-t + β2 × log10 Ei(k),j-t + β3 × Li(k),j-t + β4 × (100-Ui(k),j-t) + β5 × Pi(k),j-t +b1 × Ti(k),j-t + b 2 × log10 Ei(k),j-t + b 3 × Li(k),j-t + b 4 × (100-Ui(k),j-t) + b 5 × Pi(k),j- t + εi(k),j-t.

where i denotes the country from *i *= 1 to *i *= 119; k denotes the country from the *k *th geographic region (i.e., 16 regions based on the United Nations' classification of regions presented in Table 1); *j *is the calendar year from 1970 to 2004; *t *is the number of lag years from 0 to 10; *b0i(k) *denotes the random-effect parameter (i.e., the random intercept that estimates separate intercept values for each country); *β *0 ~ 5 denotes the fixed-effects parameters; *b0 *~ 5 denotes the random-effects parameters; and *εi(k),j-t *is the error term. In each case, the value of the time variable T was the difference between the calendar year and 1970 + *t*, since 1970 was regarded as the base year.

These linear mixed models were fitted with first-order autoregressive covariance structure matrices on the basis of Akaike's information criterion, and parameters were derived using maximum likelihood estimations. The parameter estimates of the first order autocorrelation coefficient ranged between 0.920 and 0.983, indicating a highly autoregressive correlation among the different time points of the dependent variable. Parameter estimates in the models were used to calculate the gains in life expectancy attributable to the selected socioeconomic factors. A *p *value of less than 0.05 was considered statistically significant in this study. Graphs were drawn using SigmaPlot V10.0 (Systat Software Inc., Richmond, CA, USA).

## Results

**Figure 1:**
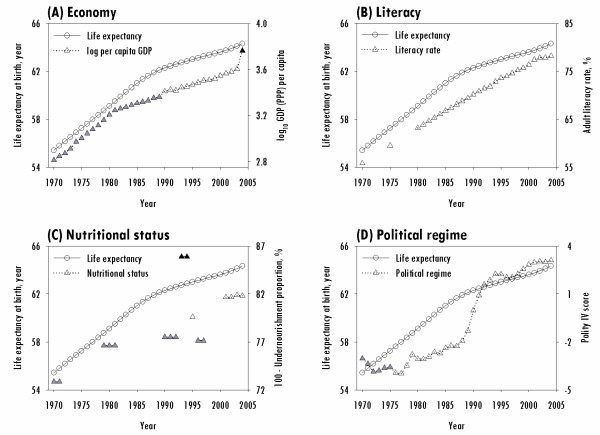
**Temporal data regarding life expectancy at birth in the context of the economy, literacy, nutritional status, and political regime from 1970 to 2004**. Symbols are drawn based on the average for countries with available data. Unshaded symbols indicate a sample that includes 100 to 119 countries. Symbols in gray represent a sample that includes 93 to 99 countries. Symbols in black indicate samples of fewer than 93 countries.

All of the factors included in the current study contributed to prolonging life expectancy, but at different magnitudes. The influence of each factor on life expectancy changed over time. Figure 1 illustrates the temporal sequences of life expectancy at birth from 1970 to 2004 in the 119 LDCs included in the study and in the context of economy, educational environment (literacy), nutritional status (undernourishment), and political regime. Panel (A) shows that life expectancy increased by 8.9 years over the 35 years. During this time period, the log per capita GDP also increased, from 2.8 in 1970 to 3.6 in 2003. Economic growth was faster before 1980 than after. Panel (B) shows a steady increase in literacy rates, with an overall increase of 22.4%, but we note that data were sparse in the 1970s. Panel (C) shows periodic data on nutritional status, with an overall decrease in undernourishment of 8.9%. Panel (D) shows that political regime scores moved slowly toward the more autocratic from 1970 to 1990 before reversing course toward the democratic direction. The scores then moved dramatically in the democratic direction from 0.9 in 1991 to 3.1 in 2004.

**Figure 2:**
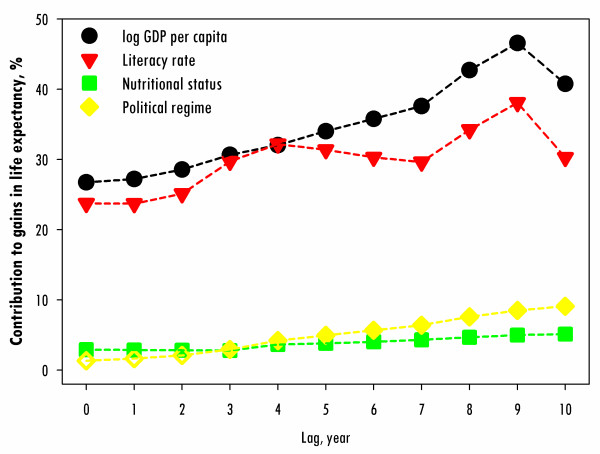
**Contribution to gains in life expectancy for 119 countries on the basis of each variable from 1970 to 2004 estimated by linear mixed models, lagged from 0 to 10 years**. Shaded symbols indicate statistically significant data in respect of the parameter estimation, while unshaded symbols are statistically non-significant.

Figure 2 shows that each of the four factors contributed between 1.34% and 46.58% of the gain in life expectancy throughout the lag periods of 0-10 years. Economy and literacy were major determinants that accounted for 26.75% - 46.58% and 23.71% - 38.08%, respectively, of the gains in life expectancy. Nutrition contributed 2.79% - 5.14% to life expectancy gains, while the political regime contributed 1.34% - 9.11%. Overall, the combined effects of these socioeconomic and political factors contributed 54.74% - 98.16% of the gain in life expectancy throughout the lag periods of 0-10 years.

Table 2 shows the modelling results of the four factors contributing to increases in life expectancy for 119 countries, with lag periods ranging from 0 to 10 years. All of the factors showed significantly positive effects on life expectancy throughout the lag periods of 0-10 years, except for the political regime score. The political regime score showed a positive effect starting at the 3rd lagged year. When comparing the standardized coefficients of the lagged effect throughout 0-10 years, economy was the most important factor, followed by literacy, nutrition, and political regime. However, when we compared the ratios of these standardized coefficients, we found that the ratios decreased over time, especially the ratios that compared political regime to the other three factors. For example, in the 1st lagged year, the standardized coefficient of economy was 166 times higher than the standardized coefficient of political regime, but by the 10th lagged year, the ratio had decreased, and the coefficient for economy was only 27 times higher.

**Table 2:** Contemporaneous and one- to 10-year lagged effects of determinants on life expectancy for 119 countries from 1970 to 2004 as estimated by linear mixed models

			Parameter estimates												
				
Models	N	*n*	***β***_**0**_	P Value	***β***_**1**_	P Value	***β***_**2**_	P Value	***β***_**3**_	P Value	***β***_**4**_	P Value	***β***_**5**_	P Value	*p*
Lag 0	119	1268	34.505	<0.001	0.147	<0.001	3.955	<0.001	0.104	<0.001	0.035	<0.001	0.017	0.155	0.983
Lag 1	118	1228	35.470	<0.001	0.142	<0.001	3.891	<0.001	0.101	<0.001	0.034	<0.001	0.020	0.107	0.982
Lag 2	116	1114	35.670	<0.001	0.131	<0.001	3.978	<0.001	0.103	<0.001	0.033	<0.001	0.025	0.069	0.978
Lag 3	116	1001	34.888	<0.001	0.105	<0.001	4.107	<0.001	0.119	<0.001	0.033	<0.001	0.034	0.028	0.972
Lag 4	116	889	33.674	<0.001	0.086	<0.001	4.218	<0.001	0.129	<0.001	0.045	<0.001	0.048	0.005	0.963
Lag 5	116	889	34.378	<0.001	0.078	0.003	4.285	<0.001	0.121	<0.001	0.044	<0.001	0.056	0.002	0.964
Lag 6	116	889	35.280	<0.001	0.072	0.007	4.298	<0.001	0.114	<0.001	0.043	<0.001	0.063	<0.001	0.965
Lag 7	116	889	36.162	<0.001	0.064	0.017	4.292	<0.001	0.107	<0.001	0.043	<0.001	0.071	<0.001	0.966
Lag 8	116	793	35.153	<0.001	0.030	0.278	4.613	<0.001	0.119	<0.001	0.043	<0.001	0.084	<0.001	0.957
Lag 9	116	697	34.049	<0.001	-0.005	0.861	4.915	<0.001	0.132	<0.001	0.045	<0.001	0.098	<0.001	0.944
Lag 10	113	582	31.609	<0.001	-0.056	0.092	5.503	<0.001	0.140	<0.001	0.058	<0.001	0.143	<0.001	0.920

N = number of countries; *n *= number of observations; *ρ *= estimate of the first order autocorrelation coefficient

## Discussion

This study contributes to the literature by quantifying the lagged effect of democracy and other socioeconomic factors on increased life expectancy over the 35 years of the study period. In our study, the four selected factors--economy, literacy, undernourishment, and political regime--together contributed 55% - 98% to the gains in life expectancy, given a lag period of up to 10 years. Improvements in a country's economy, education, and nutrition in 1 year exerted persistently positive effects on life expectancy during the subsequent 1-10 years, with the strongest effects seen in the earlier years. However, changes in political regime scores toward or away from democratic authority were more predictive of changes in life expectancy after a lag of 3+ years. The findings regarding the three socioeconomic factors were generally in agreement with past research studies [22,40-42]. These findings point out the importance of investment in economy, education, and nutrition in developing countries [43,44], and especially in Africa, where approximately one-quarter of the population still suffers from undernourishment [36].

This study's modelling results show that gains in life expectancy can be attributed more directly to improvements in the national economy than to the other factors analyzed [40,41]. Research studies have shown that improvement in life expectancy appears to have a labor productivity effect and a positive effect on economic growth [45,46]. Our study findings further point out that improving economic status may also exert a positive effect on life expectancy for several years. This finding indicates a reciprocal relationship between economy and life expectancy. Improvement on either side may eventually benefit the other.

Democracy offers health benefits, as has been seen in Soviet and Eastern European countries that experienced a transition from autocracy to democracy in the 1990s [10,47]. Our models found significant lagged effects of political regime on life expectancy, and this result is generally in line with previous findings. People living in democracies may be empowered with responsibility and awareness of their own health which could result in better health outcomes. However, the findings from the current study showed that the effect of increased democracy could take up to 3 years to manifest. What caught our attention was the magnitude of the increased influence of political regime over time, especially when compared to the influence of the three socioeconomic factors. Political regime had the least influence on increased life expectancy to begin with. Effects did not become significant until year 3, but then they continued to increase. Thus the impact of democracy on life expectancy continued to increase over time, while the impact of the three socioeconomic factors started strong but decreased over time. In each subsequent lagged year, the ratio of the standardized coefficients of the three socioeconomic factors to the standardized coefficient of political regime continued to decrease. This indicates that the relative importance of political regime to life expectancy increased over time when compared to the other factors investigated in this study. If this trend were found to continue, political regime might within two decades become a determinant as important as economy, literacy, and nutrition to increased life expectancy.

To our knowledge, this is the first study to address the longitudinal effects of political regime on life expectancy among LDCs while adjusting for time and regional correlations. Previous studies of the effects of political regime and socioeconomic factors on life expectancy were based on developed countries or on a mix of developed and developing countries [7,8,10,42]. Studies with a specific focus on developing countries have examined only single time points of data and have not considered the effects of both political regime and socioeconomic status in the analysis [7,42]. The results of our cross-country longitudinal analysis indicated that the development of public policy designed to meet social needs on improving the economy, education, and nutrition should make an important and positive contribution to population health in LDCs [7,42,44]. Most important, our findings point out that the benefits of democracy, unlike other determinants, are likely to continue to grow over time. If the identified trend is found to continue, democracy could become the most important health determinant to study in the future.

## Limitations

We extracted global data from publicly available databases to enhance data comparability with previous studies. However, the long-term data from these sources (particularly for the 1970s) are usually incomplete, and this may have affected the fitness of our linear mixed models. Having assumed that the pattern of missing data was missing at random, we applied the complete case analysis to our models. If the missing rate could be reduced, we could expect a better model fitness and parameter estimation. Another limitation of our data analysis is that we chose to render the factor scale in terms of percentage or rank constitutes. Using this scale, our models cannot identify the significant determinants of life expectancy among countries for variables with limited variation and values approaching the minima or maxima, which values were seen with both literacy and undernourishment. Population size is an important factor to be considered in evaluating population health. A weighted analysis may provide better estimation when using aggregated data from random samples of a population to make inferences at the individual level. As a weighted analysis at the country level is likely to bias results toward more populous countries, unweighted analysis has been recommended to avoid fallacy in an ecological study such as this one [48,49]. Therefore, we decided to report unweighted results as our main findings in this paper. We believe the effect of population size has been partially considered by selecting indicators representing country-level characteristics and have made inferences carefully at the country level only. Three of our five independent variables, including economy, literacy, and undernourishment, have been normalized by population for each country, while the other two variables, time and political regime, are country-level characteristics which should not be affected by population size. Hence, applying population-weighted analyses to our models will cause estimated parameters biased and unexplainable. We have actually performed WEIGHT function in the PROC MIXED procedure in our analysis, and found that these models became unstable with fluctuated (positive and negative directions) parameter estimates for the fixed effects. This indicated that normalized variables have returned to non-normalized after we weighted population-normalized variables by population size again. However, it should be noted that our findings must be cautiously interpreted within the constraints of an ecological study [48,49]. Another limitation is posed by the physical and socioeconomic determinants of population health that were not measured by our study due to the scarcity of available data. We may have overestimated the effects of significant factors in our models by not fully considering unmeasured factors such as hygiene/sanitation status, health care systems, industrialization, technological progress, natural and manmade disasters, global changes, and HIV/AIDS pandemics [47,50]. We largely assumed that our use of geographical regions would act as a proxy variable for these unmeasured variables.

In spite of these limitations, our study has several methodological strengths. Specifically, to our knowledge this study included the largest number of LDCs of any analysis to date. In addition, it included lag effects, used an estimation of random effects, and incorporated linear mixed models in the analysis. By applying time-lag effects, adjusting for random effects, and considering multiple factors in our analyses, we were able to capture 90.01%-91.42% of the variations in life expectancy seen in contemporaneous and lag models. Moreover, the random intercept in our model representing geographic heterogeneity aimed to control unstructured spatial correlations, and could result in a better parameter estimation of the studied variables. With these improvements, we were able to provide empirical evidence for how life expectancy has improved over the past few decades in 119 LDCs as a result of contemporaneous and lagged effects of economic growth, increases in literacy, nutritional improvements, and political democratization. We recommend that future campaigns to increase life expectancy adopt multifaceted approaches and consider the interplay among socioeconomic and political determinants.

## Conclusions

Though the short-term impact of democracy on increasing life expectancy is relatively small when compared to that of socioeconomic factors such as economy and nutritional status, the long-term impact of democracy increases over time and should not be underestimated. Our findings suggest, for example, that in Africa--where 35 African countries (78%) were still not categorized as democracies as of 2000-2004--any campaign to prolong life expectancies should include goals for political democratization in addition to economic development and nutritional improvements.

## Abbreviations

UN: United Nations; USCB: United States Census Bureau; GDP: Gross domestic product

## Competing interests

The authors declare that they have no competing interests.

## Authors' contributions

R-TL, Y-MC, and C-CC formulated the idea and led the study design, analysis, interpretation of the data, preparation of the manuscript, and critical revision of the manuscript for the core intellectual content. L-CC contributed to the statistical analysis and interpretation of the data. All authors contributed to discussing the content and the writing of the manuscript.

## Pre-publication history

The pre-publication history for this paper can be accessed here:

http://www.biomedcentral.com/1471-2458/12/85/prepub
